# Flat-Foot

**Published:** 1890-11-15

**Authors:** 


### SURGERY.

### FLAT-FOOT.

Our attention has lately been directed to two interesting contributions from Mr. A. G. Miller, of Edinburgh, on the subject of flat-foot. This deformity, which is also known by the names of pes planus, splay-foot, and spurious talipes valgus is often met with, especially in those who are near the boundary between the juvenile and adult stages of life. It is well defined by the author as being "mainly a giving way, or straightening out of the arch of the foot, the result of which is, that the patient when walking and leaning his weight on the foot, brings its inner as well as its outer side more or less in contact with the ground. It is a fact of common experience that in an impression on the ground of a wet healthy foot, only the toes, the heel and the outer border of the extremity are marked out. In flat-foot, especially in its most severe forms, a distinct impression is made of all parts of the sole. In almost every instance of this affection there have very probably been one or more predisposing causes in the shape of weak muscles, yielding ligaments, and imperfectly developed bones. Usually, however, some determining cause is needed to produce this unnatural condition of the foot, such cause being, as a rule, an over-straining of the ligamentary and fibrous structures. Flat foot is met with most frequently in those who walk much, or whose occupation compels them to stand for a long time, or to carry heavy weights. The tendency to depression of the inner arch of the foot is favoured by one or other of two opposed conditions, namely, by heaviness or stoutness of the patient on the one hand, and on the other by extreme weakness; the atonic type which is by far the most serious and frequently observed form of flat-foot, being generally met with, as Mr. Miller points out, in young, delicate, and often strumous-looking subjects. Flat-foot is sometimes the result of bad habit, and may occur in those who do not raise the heels in walking, it having been observed, we are told, "in butlers, and those who go about in slippers." Mr. Miller seems to attach much importance to the diagnosis of flat-foot from a somewhat similar though more severe distortion of the foot known as talipes valgus. This latter affection consists in eversion of the whole foot and is usually due to paralysis or weakness of the posterior muscles of the leg, whilst simple flat-foot is but a yielding of the inner arch of the foot and the result of stretching of ligament and fascia. It is not, however, a very easy matter to establish a clear distinction between these two forms of distortion and, indeed, some orthopædic surgeons make use of pes planus and talipes valgus as synonymous terms. Our author acknowledges that they may exist together, and there can be no doubt that in all severe and aggravated forms of flat-foot there is more or less tendency to eversion and distortion of the whole tarsus. In discussion the pathology of flat-foot, Mr. Muller in opposition to the more recent view of Von Meyer, supports the old view which is certainly still held by most orthopædic surgeons, that the giving way of the inner arch of the foot is due partly to undue straining of some of the ligaments of the tarsus and partly to weakness of the muscles which form the calf of the leg and send down their tendons to different parts of the sole. Most importance, however, is attached by Mr. Miller to the inferior calcaneo-scaphoid ligament which, passing from the os calcis to the scaphoid, supports the astragalus, and thus maintains the solidity of the inner arch of the foot. In support of the view that the calcaneo-scaphoid ligament takes the chief part in the causation of flat-foot, allusion is made to the dissections of Dr. Symington, which show that this structure is elongated in cases of the deformity, and the reader is reminded that the situations of the pain experienced in flat-foot indicate either undue stretching of the ligament, or abnormal pressure on each of the other bones which, in a sound foot, are maintained in normal relations by this strong fibrous band. If this view be a correct one, the treatment of flat-foot becomes a very simple matter, and all the surgeon has to do is to take the strain off the calcaneo-scaphoid ligament. This, of course, can be best effected by prolonged rest, but as such treatment in most instances of flat-foot would be very inconvenient and perhaps impossible, it will be found advisable to resort to some orthopædic appliance by which the strain may be taken off the inner side of the foot whilst the patient is standing or walking. The method recommended by Mr. Miller, which was first suggested by his father, Professor Miller, in 1846, is one of very easy application. This consists in making the sole of the patient's boot thicker on the inner than on the outer side, and in this way throwing the weight of the body more on the outer side of the foot. Very little importance is attached to mechanical support of the inner arch of the foot, which, it is held, in most cases only increases the patient's discomfort. Mr. Miller thinks that more can be done to effect this by improving, by means of gymnastic exercises, the tone of the posterior muscles of the leg. Mr. Miller concludes his second paper with the statement that he has not yet performed any cutting operations for flat-foot. There can be no doubt, however, that very severe and extreme forms of flat-foot do sometimes come under notice in which relief of suffering and removal of deformity can be effected by nothing short of excision of one or more of the tarsal bones.

